# Fungal Diversity and Community Assembly of Ectomycorrhizal Fungi Associated With Five Pine Species in Inner Mongolia, China

**DOI:** 10.3389/fmicb.2021.646821

**Published:** 2021-03-16

**Authors:** Yong-Long Wang, Xuan Zhang, Ying Xu, Busayo Joshua Babalola, Si-Min Xiang, Yan-Ling Zhao, Yong-Jun Fan

**Affiliations:** ^1^Faculty of Biological Science and Technology, Baotou Teacher’s College, Baotou, China; ^2^State Key Laboratory of Mycology, Institute of Microbiology, Chinese Academy of Sciences, Beijing, China; ^3^School of Life Science and Technology, Inner Mongolia University of Science and Technology, Baotou, China

**Keywords:** ectomycorrhizal fungi, pine species, fungal diversity, community assembly, host phylogeny, plant-fungi preference, stochastic processes

## Abstract

Ectomycorrhizal (EM) fungi play vital roles in ensuring host plants’ health, plant diversity, and the functionality of the ecosystem. However, EM fungal diversity, community composition, and underlying assembly processes in Inner Mongolia, China, where forests are typically semiarid and cold-temperate zones, attract less attention. In this study, we investigated EM fungal communities from 63 root samples of five common pine plants in Inner Mongolia across 1,900 km using Illumina Miseq sequencing of the fungal internal transcribed spacer 2 region. We evaluated the impact of host plant phylogeny, soil, climatic, and spatial variables on EM fungal diversity and community turnover. Deterministic vs. stochastic processes for EM fungal community assembly were quantified using β-nearest taxon index scores. In total, we identified 288 EM fungal operational taxonomic units (OTUs) belonging to 31 lineages, of which the most abundant lineages were *Tomentella–Thelephora*, *Wilcoxina*, *Tricholoma*, and *Suillus–Rhizopogon*. Variations in EM fungal OTU richness and community composition were significantly predicted by host phylogeny, soil (total nitrogen, phosphorus, nitrogen–phosphorus ratio, and magnesium), climate, and spatial distance, with the host plant being the most important factor. β-nearest taxon index demonstrated that both deterministic and stochastic processes jointly determined the community assembly of EM fungi, with the predominance of stochastic processes. At the Saihanwula site selected for preference analysis, all plant species (100%) presented significant preferences for EM fungi, 54% of abundant EM fungal OTUs showed significant preferences for host plants, and 26% of pairs of plant species and abundant fungal OTUs exhibited remarkably strong preferences. Overall, we inferred that the high diversity and distinctive community composition of EM fungi associated with natural pine species in Inner Mongolia and the stochastic processes prevailed in determining the community assembly of EM fungi. Our study shed light on the diversity and community assembly of EM fungi associated with common pine species in semiarid and cold temperate forests in Inner Mongolia, China, for the first time and provided a better understanding of the ecological processes underlying the community assembly of mutualistic fungi.

## Introduction

The long-standing principle of community ecology is to examine the mechanisms underlying biodiversity conservation and community assembly (Chase, 2003; Nemergut et al., 2013; Zhou and Ning, 2017; Ladau and Eloe-Fadrosh, 2019). As an integral part of soil microorganisms, ectomycorrhizal (EM) fungi often establish mutual symbionts with many woody plants. For instance, they play vital roles in facilitating host plant fitness, forest biodiversity, and global nutrient cycling (Smith and Read, 2008; Tedersoo et al., 2020). Therefore, clarifying the underlying factors of EM fungi community assembly could vastly improve our predictability in the areas of sustainable forests, biochemical dynamics, and ecological processes under severe global changes.

Recent studies have emphasized that microbial communities are affected mainly by deterministic (niche theory) and stochastic processes (neutral theory) (Stegen et al., 2012; Nemergut et al., 2013; Bahram et al., 2018; Wang et al., 2020). The niche theory emphasizes that the species are under ecological selection pressure imposed by biotic and abiotic factors due to different habitats preference and fitness (Dumbrell et al., 2009; Stegen et al., 2012); neutral theory asserts the role of dispersal limitation, ecological drift, and diversification in predicting community assembly independent of ecological selection (Hubbell, 2001). It is widely accepted that deterministic and stochastic processes can jointly regulate microbial community assembly (e.g., Dumbrell et al., 2009; Wang et al., 2020), and the effect of the deterministic factors in describing the community assembly of EM fungi has been previously investigated. For example, host phylogeny was the most important determinant of the development of EM fungal communities associated with Betulaceae and Fagaceae species in Chinese forest ecosystems (Wu et al., 2018; Wang Y.L. et al., 2019) and Salicaceae species in Estonia (Tedersoo et al., 2013). Host plants can significantly affect EM fungal communities through host specificity/preference (Tedersoo et al., 2010a; van der Linde et al., 2018; Wang Y.L. et al., 2019) and modified rhizosphere microhabitats (Aponte et al., 2010). In comparison, some studies have shown that the host influence is absent or minor (Glassman et al., 2015, 2017; Miyamoto et al., 2015). A growing body of evidence indicates that phylogenetic analysis is a more effective technique than the use of plant identity to detect the host effect, as phylogenetic eigenvectors can represent different divisions at different taxonomic levels between host plants (Tedersoo et al., 2013). Similarly, abiotic environmental factors, including soil and climatic variables, have been reported as strong drivers of the EM fungal communities; for instance, climate difference and soil nutrients explained the largest variations of EM fungal community in two Japanese mountains (Miyamoto et al., 2015) and seven willow species in southeastern Minnesota, United States (Erlandson et al., 2016), respectively. In general, the effect of the deterministic process in defining the EM fungal community could be attributed to the selection of biotic and abiotic environmental variables on EM fungi through their fitness in response to the surrounding conditions (De Wit and Bouvier, 2006).

Stochastic processes such as dispersal limitation have been reported as an important driver of EM fungal community assembly (Glassman et al., 2015; Wu et al., 2018; Wang Y.L. et al., 2019). The reasons for dispersal limitation fall into two categories. On the one hand, fungal species differ in the characteristics associated with dispersal, such as spore size and yield, resulting in different dispersal abilities among fungal species (Peay et al., 2012; Kivlin et al., 2014; Peay and Bruns, 2014); on the other hand, the geographic distance could impede the movement of fungal individuals from one habitat to new suitable ones. Thus, these two factors contribute to the effect of dispersal limitation on the turnover of the EM fungal community. Several studies have shown that the geographic distance is a better indicator for the EM fungal community than other variables such as host plant, soil, and climatic parameters; for example, geographic distance explained the greatest variation in the EM fungal community associated with pine species across North America (Glassman et al., 2015).

The essential or best predictor variables responsible for the turnover of the EM fungal communities varied among studies. This inconsistency may have resulted from different spatial scales, forest types, and environmental variables. Previous studies have concentrated on EM fungal communities in subtropical, tropical, and temperate monsoon forest habitats where high biodiversity is present (Tedersoo et al., 2008, 2010a; Gao et al., 2015; Matsuoka et al., 2016). However, less attention has been given to EM fungi in semiarid and arid forests and cold temperate forests. Thus, EM fungal diversity and composition, the relative importance of host plant, soil, climate, and geographic distance on the EM fungal community, and the underlying ecological process driving EM fungal community assembly (stochastic and deterministic processes) in these forest ecosystems remain poorly understood.

The forests in Inner Mongolia, China, located in the semiarid and cold temperate zones, have relatively less precipitation and lower temperature when compared with subtropical, tropical, and warm forests. In the forests of Inner Mongolia, China, pine species are the most common woody plants, making a major ecological and economic contribution to the biodiversity of forests and humans, such as nuts and timber. However, pine-associated EM fungal diversity, community composition, and underlying assembly mechanisms in Inner Mongolia remain largely undefined. Here, we have identified the EM fungal community associated with pine species in Inner Mongolia, China, and we especially aimed to: (1) completely unveil EM fungal diversity and composition; (2) identify major factors influencing the diversity and composition of the EM fungi and evaluate the relative importance of deterministic and stochastic processes in shaping EM fungal community assembly; and (3) investigate the preference of the host and fungi in plant–EM fungal interactions. Thus, we assessed the EM fungal community of five common pine species in secondary forests in Inner Mongolia over approximately 1,900 km, using the Illumina Miseq sequencing of the internal transcribed fungal spacer 2 (ITS2). We also established the major drivers responsible for the shift in the EM fungal community by analyzing the impact of host plants, soil, climatic variables, and spatial distance, and we investigated the relative importance of deterministic and stochastic processes in shaping the EM fungal community assembly. We hypothesized that (1) host plant, abiotic variables (i.e., soil and climate), and geographic distance all play significant roles in determining EM fungal community, but the host plant should be a stronger driver than other predictors; (2) significant partner preference should be present in pine–EM fungal associations; and (3) deterministic and stochastic processes concurrently control the community assembly of EM fungi.

## Materials and Methods

### Sampling

We selected eight typical secondary forests, approximately 60 years old and with no history of disturbance, during the peak period of the growing season (Supplementary Figure 1). The selected forests were located in semiarid and cold temperate zones, with mean annual temperature (MAT) ranging from −4.75 to 5.97°C and mean annual precipitation (MAP) from 267 to 506 mm according to the climatic information extracted from the WorldClim dataset at 30-arc-second resolution (Hijmans et al., 2005). Pine species, with very few other EM hosts (Betulaceae and Salicaceae plants), were the dominant woody plants in each forest. At each site, 4–7 individuals were selected from each pine species. The fine roots were excavated by tracing the roots to the base of the trunk of every single plant. At each site, a sample of rhizosphere soil was collected from each individual and then combined as one composite sample. The individuals were at least 10 m apart to ensure the independence of the samples (Pickles et al., 2012). Root samples were transported to the laboratory in an icebox and kept at −20°C before processing. Soil samples were air-dried, sieved through 2 mm mesh, and then used for property analysis. A total of 63 root samples and eight soil samples were obtained in this study. Geographical coordinates and latitude were recorded using a high-sensitivity Global Positioning System instrument (M-241, Holux Technology Inc., Taiwan, China). Climatic conditions, including MAT and MAP of each site, were compiled from the WorldClim dataset at 30-arc-second resolution (Hijmans et al., 2005). Information on the geographic locations and climate is summarized in Supplementary Table 1.

### Analysis of Soil Properties

Total nitrogen (N) was quantified by adopting the semi-micro-Kjeldahl process. Total phosphorus (P) was measured using iCAP 6300 inductively coupled plasma spectrometers (Thermo Science, Wilmington, United States). Total calcium (Ca) and magnesium (Mg) were measured in ICP Optima 8000 (Perkin-Elmer, Waltham, MA, United States). Information on soil properties can be found in Supplementary Table 1.

### DNA Extraction, PCR, and MiSeq Sequencing

Before molecular analysis, the EM root tips were first detected and picked under a stereomicroscope. A total of 200 healthy EM root tips were randomly selected from each plant, resulting in 12,600 EM root tips from 63 samples used for DNA extraction. High throughput sequencing is becoming more popular in EM fungal community studies (e.g., Matsuoka et al., 2016; Van Geel et al., 2018; Wu et al., 2018; Wang Y.L. et al., 2019). Importantly, high throughput sequencing data can reflect the real dominant or rare taxa based on the relative sequencing abundance, whereas Sanger sequencing data present the frequency of EM root tips in samples. Thus, the method of high throughput sequencing based on the mixture of EM root tips was adopted in our study. The detailed process was as follows: the total genomic DNA of each sample was extracted using the modified cetyltrimethylammonium bromide method described in Gao et al. (2013). A two-step polymerase chain reaction (PCR) procedure has been adopted to amplify the fungal ITS2 region in the Veriti 96-well Thermal Cycler (Applied Biosystems, Foster City, United States). Briefly, the primers ITS1f (Gardes and Bruns, 1993) and ITS4 (White et al., 1990) were used to amplify the entire ITS region in 25 μl of reaction mixtures, including 1-U KOD-Plus-Neo DNA polymerase (Toyobo, Osaka, Japan), 2.5 μl of 10 × buffer, 2 mM of each deoxyribonucleotide triphosphate, 25 mM of magnesium sulfate, 10 μM of each primer, and approximately 5 ng of the DNA template. The PCR temperature profile included initial denaturation at 95°C for 5 min, 20 cycles at 94°C for 1 min, 58°C for 50 s, and 68°C for 30 s, and then final extension at 68°C for 10 min. The PCR products were diluted 30 times, and 1.5 μl solution was used as a template for the second PCR amplification. Primers fITS7 (Ihrmark et al., 2012) and ITS4 with unique barcode tags (12-base barcode sequence) were adopted to target the ITS2 region, and the PCR conditions were similar to the first amplification. Three replicates were used for each sample and then pooled to represent that sample. The pooled PCR products of each sample were purified using the Wizard SV Gel and the PCR Clean-Up System (Promega, Madison, United States). All purified PCR products were pooled with equimolar amounts and then sequenced to the Illumina MiSeq PE250 (Illumina, San Diego, CA, United States) platform at the Chengdu Institute of Biology Environmental Genome, Chinese Academy of Sciences, China.

### Bioinformatic Analysis

Raw sequence data were filtered using the Quantitative Insights in Microbial Ecology platform (Caporaso et al., 2010) to exclude low-quality reads to those with uncertain bases >6, uneven to the primer or barcode sequence, duration <250 bp, or average quality score <20. The ITS2 region of the filtered sequences was extracted using the ITSx software (Bengtsson-Palme et al., 2013), and the possible chimera sequences were identified using the chimera.uchime command in Mothur 1.31.2 (Schloss et al., 2009) by referring to the entries in the unified DNA-based fungal species database (UNITE) (Kõljalg et al., 2013) and then discarded. After this step, the remaining sequences were clustered into operational taxonomic units (OTUs) at a 97% similarity threshold using the UPARSE pipeline (Edgar, 2013), during which singletons were also deleted. Using the simple local alignment search tool (BLAST) (Altschul et al., 1990), the representative sequence (most abundant) of each OTU was searched against the UNITE database (v. 8.2, release date: 02.04.2020). Fungal OTUs were first identified and assigned to taxonomic identity on the basis of the criteria proposed by Tedersoo et al. (2014), then EM fungal OTUs were identified on the basis of Tedersoo and Smith (2013) and Tedersoo et al. (2014) if they better matched the known EM fungal taxa and lineages. To eliminate the effect of different sequencing depths across samples on the subsequent analysis, the EM fungal data were normalized to 1,055 (the smallest sample size) using the subsample command in Mothur. The representative sequences of each EM fungal OTU were deposited in the European Nucleotide Archive under study accession no. LR989273-LR989560. Information on EM fungi in the present study is shown in Supplementary Table 2.

### Statistical Analysis

All statistical analyses were conducted in R 3.6.2 (R Development Core Team, 2019). The MaturaseK gene (matK) exists in chloroplasts, which is highly conserved in plant systematics, and is widely used in plant phylogeny analyses (Hilu and Liang, 1997; Cheng et al., 2000; Wang et al., 2000). Thus, the matK gene of the five pine species in the present study (Supplementary Table 3) was downloaded from the National Center for Biotechnology Information to create the host plant phylogenetic eigenvector used for the subsequent analysis, as other genes such as ITS were not fully accessible and the matK gene reflects the phylogeny of the pine species in this study. In MAFFT v. 7.215 (Katoh and Standley, 2013), the plant matK sequences were first aligned using the L-INS-i algorithm, and then a maximum likelihood phylogeny tree was created with 1,000 bootstrap replicates under the GTR model in MEGA ver 7.0 (Kumar et al., 2016; Supplementary Figure 2). After this, the phylogenetic distances between each plant species in the phylogenetic tree were calculated using the cophenetic.phylo command in the ape package (Paradis et al., 2004), and then the principal coordinate analysis (PCoA) was used to transform the pairwise distance matrix into phylogenetic eigenvectors using the cmdscale command in the vegan package (Oksanen et al., 2013). Similarly, the geographic distance between each site was first calculated on the basis of the geographic coordinates, and the resulting distance matrices were transformed into spatial eigenvectors of the principal coordinate of neighbor matrices (PCNM) using the pcnm command in the PCNM package (Dray et al., 2006), and only those with positive eigenvalues were retained for the subsequent analysis.

EM fungal OTU accumulation curves for each plant species were drawn using the specaccum command in the vegan package (Oksanen et al., 2013). The non-parametric Kruskal–Wallis test was used to determine the difference in EM fungal OTU richness between five plant species, as the data did not satisfy homogeneity of variance before and after logarithmic and square root transformation, and then the Conover test was used to conduct multiple comparisons between each plant species using the *post hoc*.kruskal.conover.test function in the PMCMR package (Pohlert, 2014). The Venn diagram was created to examine the shared and unique EM fungal OTUs among the five plant species using the online software E-venn^1^.

Krona (v.2.6, Ondov et al., 2011) was adopted to visualize the taxonomic composition of EM fungi through the Krona pie chart. A Bray–Curtis dissimilarity matrix for fungal communities (Hellinger-transformed data) was calculated using the vegdist command in the vegan package (Oksanen et al., 2013). To measure the difference between the EM fungal communities of the plant species, the communities were ordinated by non-metric multidimensional scaling (NMDS) analysis through the use of the metaMDS command in the vegan package (Oksanen et al., 2013), and the confidence intervals of samples of each plant species were drawn from the NMDS ordination using the ordiellipse function. The significance of the difference between the five plant species in the fungal communities was assessed on the basis of the permutational multivariate variance analysis (PerMANOVA) by using the adonis command in the same package. Meanwhile, permutation analysis of multivariate dispersion (PERMDISP) (Anderson, 2001, 2006) was conducted to test whether groups differed in their dispersion by using the betadisper function in the vegan package (Oksanen et al., 2013). Reanalyses of NMDS, PerMANOVA, and PERMDISP were performed on the SHWL site data to confirm the host effect on EM fungal community. To further evaluate the variation of the relative abundances of dominant EM fungal lineages (>5% of total sequences) across different plant species, a non-parametric Kruskal–Wallis was used, as the data did not follow the normality and homogeneity of the variance, and the Conover test was used to conduct multiple comparisons as described earlier. To identify the key factors affecting the composition of the EM fungal community, the host phylogenetic, spatial, soil, and climatic variables were fit onto the ordination plot based on the environmental fitting test using the envfit command in the vegan package (Oksanen et al., 2013). Additionally, random forest modeling was adopted to identify the main drivers of the EM fungal OTU richness and community composition using the randomForest command in the randomForest package (Liaw and Wiener, 2002). The importance and significance of each predictor in response variables were evaluated using rfPermute and rp.importance commands in the rfPermute package (Archer, 2013), respectively. Mantel and partial mantel tests were conducted to determine the correlations between the EM fungal communities and the host phylogenetic and geographic distance using the mantel command in the vegan package (Oksanen et al., 2013). To assess the potential effect of dispersal limitation on the fungal community assembly, the Sloan neutral model was adopted by evaluating the relationship between the frequency of occurrence of OTUs and their relative abundance (logarithmic transformed) based on a parameter describing the migration rate “m” of the individual fungal OTUs (Sloan et al., 2006). The estimated “m” parameter indicates the probability that a random loss of an individual OTU from a local community would be replaced by dispersal from the metacommunity and, therefore, could be used as a dispersal limitation measure. The lower value of “m” suggests more limited dispersal in the turnover of the community.

Using phylogenetic information and associated randomization procedures to investigate underlying ecological processes for microbial community assembly has become popular in recent years (Stegen et al., 2012, 2013; Wang et al., 2020). To evaluate phylogenetic community composition, the mean nearest taxon distance (MNTD) and the nearest taxon index (NTI) were calculated using the mntd and ses.mntd functions in the picant package (Kembel et al., 2010). The MNTD measures the mean nearest taxon distance between all pairs of OTUs in one sample, and the NTI measures the standard deviation of observed MNTD compared with randomized MNTD (Webb et al., 2002). When the NTI >2 in a single community or the mean NTI of all communities >0 significantly means taxa in the communities are more closely related than expected by chance, indicating phylogenetic clustering. When the NTI <−2 or mean NTI <0 significantly, this suggests phylogenetic over-dispersion and that species in the communities were distantly related. Additionally, if phylogenetic relatedness was not significantly different from null models, it indicates the predominance of stochastic processes. The significance was evaluated based on the one-sample *t*-test. To investigate the assembly process of the EM fungal community, phylogenetic turnover between communities was adopted in our study according to the framework proposed by Stegen et al. (2013) in which β-nearest taxon index (βNTI) was evaluated. Briefly, pairwise phylogenetic turnover among communities was quantified based on the MNTD metric (βMNTD) by using the comdistnt function in the picante package (Kembel et al., 2010) (abundance. weighted = TRUE). Then, the taxa labels of the phylogenetic tree were shuffled 999 times to generate null distributions of βMNTD among samples. The standard effect size of βMNTD (i.e., βNTI) was obtained based on the number of standard deviations that the observed βMNTD is from the mean of the null distribution. | βNTI| >2 means that phylogenetic turnover is significantly greater (lower) compared with null distribution, implying the dominance of deterministic process, by contrast, when | βNTI| <2, it suggests phylogenetic turnover is not significantly different from null expectation, indicating the important role of stochastic process in community assembly.

To evaluate the preference of the plant–fungal association and to eliminate the influence of the site on the analysis, the Saihanwula (SHWL) site, where the number of plant species was three, was selected for the preference analysis. Briefly, preference analysis evaluates the host and fungal preferences for their partners and two-dimensional preferences in pairs of plant species and fungal OTUs. The calculation of the relevant index was based on the presence/absence dataset by using original and randomized matrices. The detailed procedures have been described in Toju et al. (2016) and Wang Y.L. et al. (2019).

## Results

### Ectomycorrhizal Fungal Database Summary

After quality filtering, a total of 969,290 non-chimeric ITS2 sequences were generated from 1,017,759 raw sequences, and these high-quality sequences were clustered into 1,734 non-singleton OTUs (951,673), of which 349 (750,517) were identified as EM fungal. After the rarefication, a normalized dataset of 288 EM fungal OTUs was retained for further analysis. The 30 most abundant OTUs occupied 70% of the overall sequences, and 139 of 288 OTUs (48%) occurred in fewer than three samples (Supplementary Figure 3).

### Ectomycorrhizal Fungal Diversity

**FIGURE 1:**
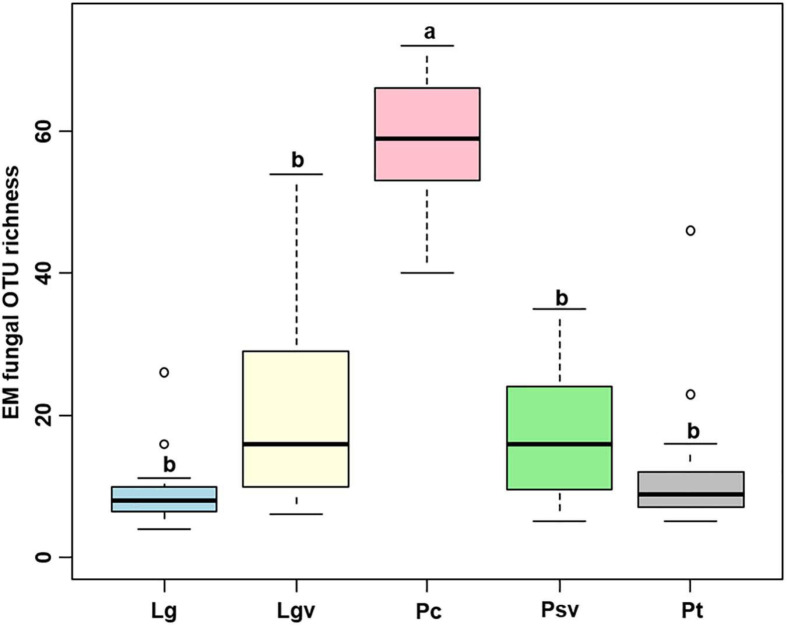
Richness of ectomycorrhizal (EM) fungal operational taxonomic units (OTUs) in the five pine species. Bars without shared letters indicate significant differences in richness of fungal OTUs among the five plant species, according to Conover’s test.

**FIGURE 2:**
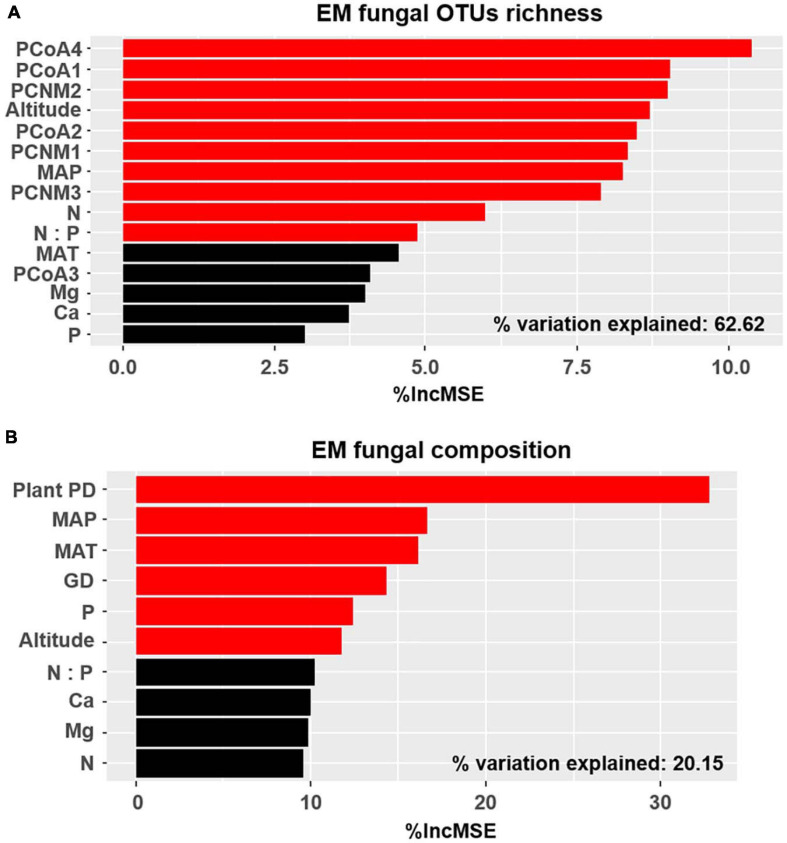
Random forest model demonstrating relative importance of host plants, spatial, soil, and climatic variables for ectomycorrhizal (EM) fungal OTU richness **(A)** and community composition **(B)** %IncMSE, % of increase of mean square error; PCoA, principal coordinate analysis of host phylogeny; PCNM, principal coordinates of neighbor matrices; MAT, mean annual temperature; MAP, mean annual precipitation; P, soil total phosphorus; N, soil total nitrogen; Ca, soil total calcium; Mg, soil total magnesium; PD, phylogenetic distance; GD, geographic distance; Significant factors are shown in red (*P* < 0.05) and non-significant factors are shown in black (*P* > 0.05).

The accumulation curves of each plant species did not show any signs of reaching an asymptote, suggesting that further sample collection may result in more unknown EM fungal OTUs (Supplementary Figure 4). The Kruskal–Wallis test showed that the EM fungal OTU richness was significantly different between plant species, and the multiple comparisons suggested that the fungal OTU richness of *Picea crassifolia* was significantly higher than that of the other four plant species (Figure 1). Random forest analysis indicated host phylogeny (PCoA1, 2, and 4), spatial distance (PCNM1, 2, and 3), MAP, soil N, N:P, and altitude significantly affected EM fungal OTU richness, whereas 62.62% of the richness variance was explained by the model (Figure 2A).

### Ectomycorrhizal Fungal Community Composition and Assembly

**FIGURE 3:**
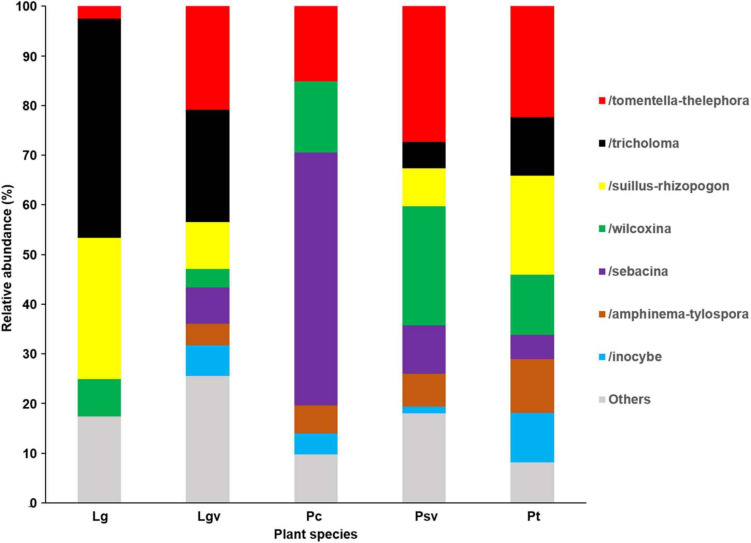
Ectomycorrhizal fungal lineage and their relative abundance in five pine species. Here, we only showed abundant lineages (>5% of total sequences). Lg, *Larix gmelinii*; Lgv, *Larix gmelinii* var. principis-rupprechtii; Pc, *Picea crassifolia*; Psv, *Pinus sylvestris* var. mongolica; Pt, *Pinus tabuliformis*.

**FIGURE 4:**
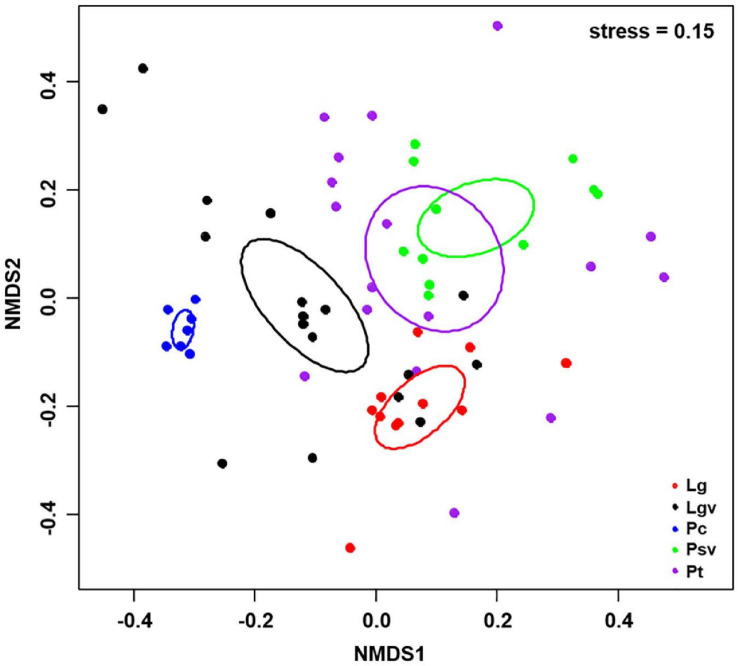
Non-metric multidimensional scaling (NMDS) ordination of ectomycorrhizal fungal community composition based on Bray–Curtis distance (stress = 0.15). Ellipses delimit 95% confidence intervals around centroids for each pine species. Lg, *Larix gmelinii*; Lgv, *Larix gmelinii* var. principis-rupprechtii; Pc, *Picea crassifolia*; Psv, *Pinus sylvestris* var. mongolica; Pt, *Pinus tabuliformis*.

**FIGURE 5:**
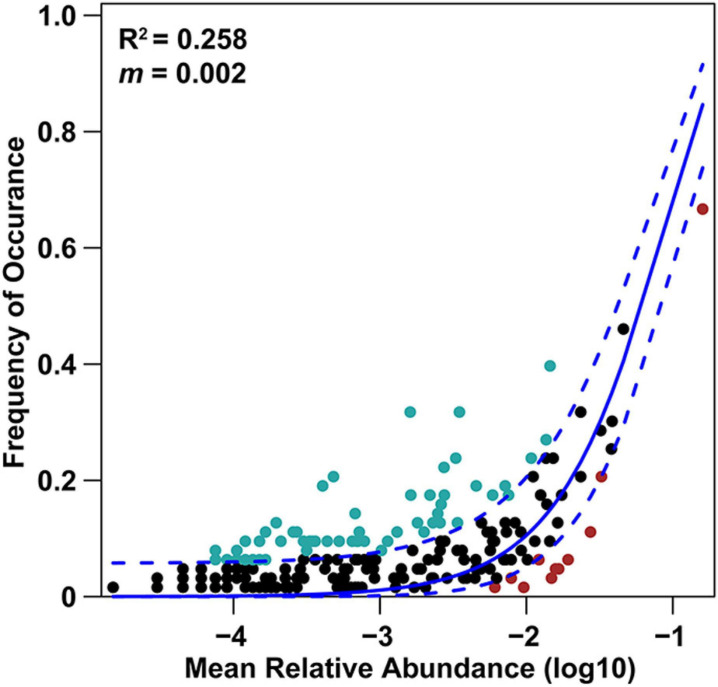
Fit of neutral model for fungal OTUs. Solid blue line represents the predicted occurrence frequency, and dashed blue lines represent 95% confidence intervals around the model prediction; light blue and red dots indicate the OTUs that occur more and less frequently than given by the model; R^2^ indicates the fit to the neutral model; m indicates immigration rate.

**FIGURE 6:**
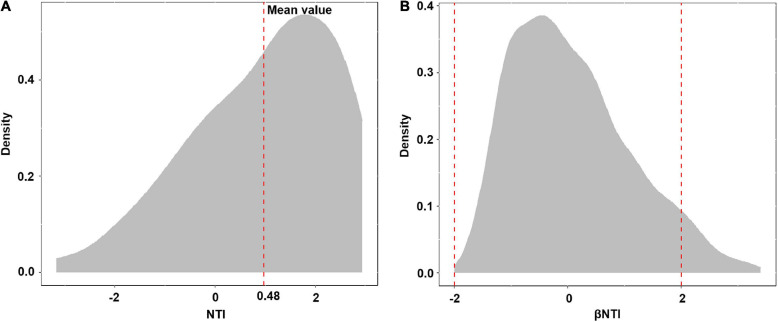
Distribution of nearest taxon index (NTI) **(A)** and beta nearest taxon index (βNTI) **(B)**.

Basidiomycota dominated 83% of total EM fungal reads, whereas Ascomycota only occupied 13% (Supplementary Figure 5). A total of 31 lineages were found in 63 root samples of five pine species in the current study, of which *Tomentella–Thelephora*, *Wilcoxina*, *Tricholoma*, *Suillus-Rhizopogon*, *Sebacina*, *Amphinema-Tylospora*, and *Inocybe* were the most abundant EM fungal lineages, accounting for 83.64% of total sequences (Table 1). The relative abundance of the seven abundant EM fungal lineages in each plant species is shown in Figure 3. The NMDS ordination showed that the EM fungal community was significantly different among the plant species (Adonis: *R*^2^ = 0.234, *P* = 0.001, Figure 4), whereas the PERMDISP that indicated the dispersion among groups (plant species) differed significantly (*F* = 9.42, *P* < 0.001). Repeating the analysis of NMDS, PerMANOVA, and PERMDISP on the SHWL dataset indicated that the EM fungal communities were significantly among plant species (*R*^2^ = 0.37, *P* = 0.001, Supplementary Figure 6), and the dataset did not vary significantly in dispersion by groups (*F* = 2.57, *P* = 0.12). The environmental fitting test indicated that host phylogeny (PCoA1 and 4), spatial distance (PCNM1 and 2), MAT, MAP, soil N, P, N:P, Mg, and altitude were significantly correlated with the EM fungal community (Supplementary Figure 7). Similarly, random forest analysis showed that plant phylogenetic distance, spatial distance, MAT, MAP, soil P, and altitude were the most significant determinants of dissimilarity in the EM fungal community, and the variation explained by the model was 20.15% (Figure 2B). Mantel and partial mantel analyses indicated that the EM fungal community was significantly correlated with host phylogenetic distance (Mantel *r* = 0.335, *P* = 0.001; partial Mantel *r* = 0.228, *P* = 0.001) and geographic distance (Mantel *r* = 0.260, *P* = 0.001; partial Mantel *r* = 0.099, *P* = 0001) (Supplementary Figure 8). The neutral model suggested dispersal limitation significantly affected the assembly of the EM fungal community (*R*^2^ = 0.258, *m* = 0.002, Figure 5). The NTI values ranged from −1.58 to 1.47, and the mean NTI value across all samples was not significantly different from 0 (*P* > 0.05), indicating the phylogenetic relatedness in the communities was stochastic (Figure 6A). Most βNTI values fell within the range of −2 to 2, with βNTI >2 existing in only 5% of the cases, indicating that stochastic processes had a stronger effect on the community assembly of EM fungi than the deterministic processes in the present study (Figure 6B).

**TABLE 1:** Relative abundance of each ectomycorrhizal (EM) fungal lineage in each plant species.

EM fungal lineage	Lg (%)	Lgv (%)	Pc (%)	Psv (%)	Pt (%)	Total (%)
*/Tomentella–Thelephora*	2.47	20.89	15.11	27.32	22.37	18.55
*/Tricholoma*	44.21	22.61	−	5.29	11.80	17.93
*/Suillus–Rhizopogon*	28.43	9.36	0.04	7.63	19.93	14.20
*/Wilcoxina*	7.46	3.75	14.30	24.07	12.07	11.36
*/Sebacina*	0.04	7.33	50.91	9.75	4.90	10.67
*/Amphinema–Tylospora*	0.02	4.32	5.62	6.57	10.83	5.86
*/Inocybe*	0.01	6.21	4.25	1.34	9.94	5.06
*/Piloderma*	13.04	6.90	−	0.32	−	4.19
*/Tuber–Helvella*	0.57	0.94	0.66	7.65	2.29	2.38
*/Russula–Lactarius*	−	5.53	−	2.87	0.40	2.10
*/Geopora*	0.49	0.57	0.16	1.73	4.62	1.81
*/Cortinarius*	0.01	4.63	0.83	−	0.01	1.35
*/Clavulina*	−	4.50	−	−	0.02	1.22
*/Helotiales*	0.40	0.09	5.74	0.03	0.07	0.76
*/Hygrophorus*	0.05	0.01	−	3.30	0.07	0.61
*/Cenococcum*	−	0.35	0.35	0.96	0.33	0.39
*/Terfezia–Peziza depressa*	1.37	0.01	−	0.51	0.06	0.35
*/Pseudotomentella*	1.40	0.16	−	0.01	−	0.29
*/Serendipita*	−	0.48	−	0.06	−	0.14
*/Tomentellopsis*	−	0.48	0.05	−	−	0.14
*/Cantharellus*	−	−	1.21	−	−	0.13
*/Hebeloma–Alnicola*	0.02	0.35	0.08	0.07	−	0.12
*/Genea–Humaria*	0.02	−	−	0.43	−	0.08
*/Otidea*	−	0.01	0.66	−	−	0.08
*/Tarzetta*	−	−	−	−	0.28	0.08
*/Boletus*	−	0.26	−	−	−	0.07
*/Serendipita*	−	0.21	−	0.01	−	0.06
*/Meliniomyces*	−	0.04	−	0.09	0.01	0.03
*/Ramaria-Gautieria*	−	0.03	−	−	−	< 0.01
*/Elaphomyces*	−	−	0.01	−	−	< 0.01
*/Laccaria*	−	−	−	−	0.01	< 0.01

*Lg, Larix gmelinii; Lgv, Larix gmelinii var. principis-rupprechtii; Pc, Picea crassifolia; Psv, Pinus sylvestris var. mongolica; Pt, Pinus tabuliformis.*

### Plant–Fungus Preference

**FIGURE 7:**
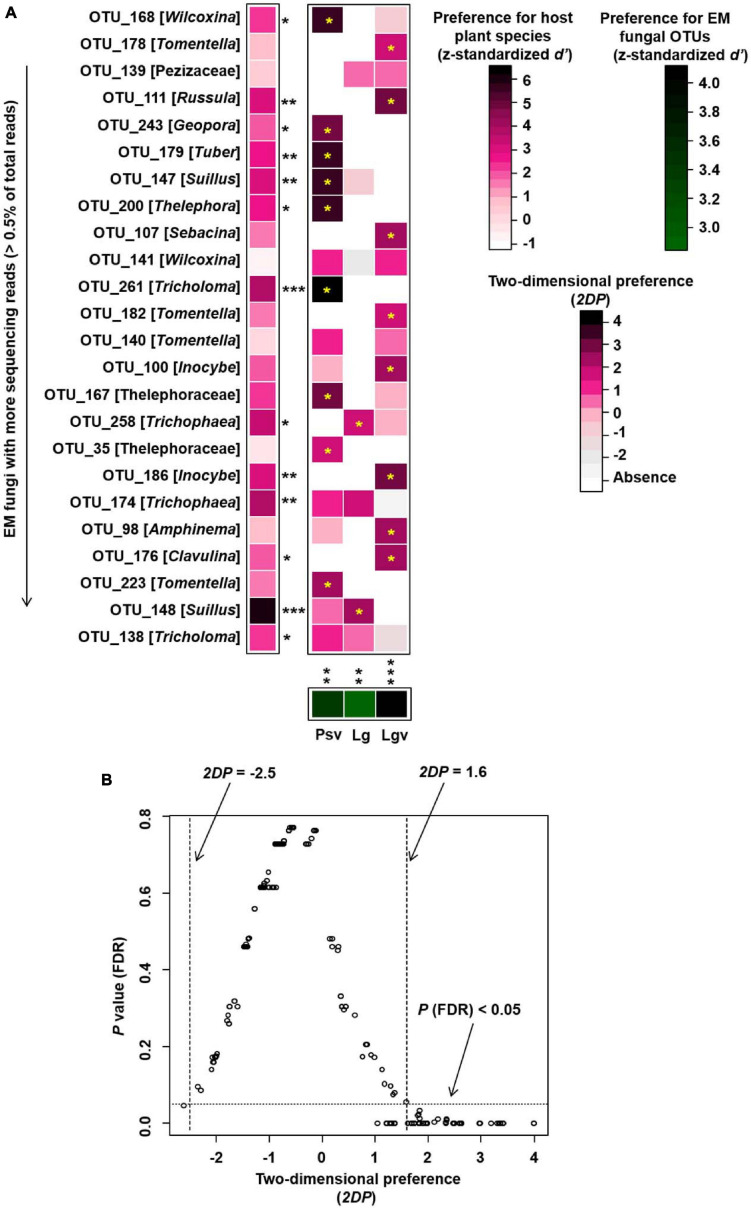
Preferences observed in plant–fungus associations in Saihanwula. **(A)** standardized *d’* estimates of preferences for ectomycorrhizal (EM) fungal operational taxonomic units (OTUs) for indicated plant species (columns). Likewise, the standardized *d’* estimate of preferences for plant species is indicated for each of the observed fungal OTUs (row). A cell in the matrix indicates a two-dimensional preference (*2DP*) estimate, indicating the extent an association of a focal plant–fungus pair was observed more/less frequently than expected by chance. Cell with an asterisk inside represents significant preferences in plant–fungus pair. Because multiple species/OTUs were tested, the *P*-values are shown as false discovery rates (FDRs) in the plant/fungus analysis. **(B)** Relationship between *2DP* and FDR-adjusted *P*-values, *2DP* values larger than 1.6 represented strong preferences. Significance: *P < 0.05, **P < 0.01, ***P < 0.001. Psv, *Pinus sylvestris* var. mongolica; Lg, *Larix gmelinii*; Lgv, *Larix gmelinii* var. principis-rupprechtii.

Preference analysis conducted at SHWL indicated that 13 out of 24 (54%) abundant EM fungal OTUs were significantly preferred to specific plant species, and all plant species (100%) showed a significant preference for some EM fungal OTUs; in addition, 19 out of 72 (26%) pairs of plant species and abundant fungal OTUs exhibited remarkably strong preferences (Figure 7).

## Discussion

A total of 288 EM fungal OTUs belonging to 31 lineages were identified in this analysis, with a richness value of 17.49 ± 2.20 (mean ± SE) across all samples. The species rarefaction curve did not reach the plateau, implying our sample size was not adequate. Thus, more samples should be collected in our future study, which may introduce higher fungal diversity. Here, we found that the richness of EM fungal OTUs was significantly affected by host phylogeny, spatial distance, MAP, soil N, N:P ratio, and altitude. This was consistent with the reports in many previous studies (Põlme et al., 2013; Tedersoo et al., 2013; Wu et al., 2018; Wang Y.L. et al., 2019). For example, host phylogenetic eigenvectors were the significant variables responsible for EM fungal OTU diversity in the studies involved in Betulaceae, Fagaceae, and Salicaceae plants (Tedersoo et al., 2013; Wu et al., 2018; Wang Y.L. et al., 2019). These studies combined with our study indicated that host plants play important roles in determining EM fungal diversity and further support the hypothesis that host taxonomic relatedness governs the “host effect” (Taylor, 2008; Tedersoo et al., 2013). The influence of MAP and soil nutrients on EM fungal richness is mainly because EM fungi should grow under suitable habitat conditions; indeed, previous studies have found that high precipitation and increased N supply could reduce EM fungal diversity (Cox et al., 2010; Tedersoo et al., 2013).

In our study, the dominant EM fungal lineages were *Tomentella–Thelephora*, *Tricholoma*, *Suillus–Rhizopogon*, *Wilcoxin*, *Sebacin*, *Amphinema–Tylospora*, and *Inocybe*, and lineages accounted for 83.64% of total sequences. The most abundant lineage was *Tomentella–Thelephora*, which was consistent with many previous studies involving various host plant groups investigated in different forest ecosystems (Tedersoo et al., 2013; Wang et al., 2017; Wu et al., 2018; Wang Y.L. et al., 2019) and in line with Tedersoo et al. (2010b) that indicated *Tomentella–Thelephora* was a globally distributed lineage. However, the dominance of lineages *Tricholoma*, *Suillus–Rhizopogon*, *Wilcoxin*, and *Amphinema–Tylospora* has rarely been recorded. For example, *Tricholoma* accounted for 17.93% of total sequences and occurred in 44 of 63 samples (69.84%), suggesting that this lineage was a widespread lineage associated with pine species in Inner Mongolia. Additionally, *Amphinema–Tylospora* has been reported as a Pinaceae-specific lineage in previous studies (Miyamoto et al., 2020; Vlk et al., 2020), and *Suillus* was found to be the dominant genus associated with *Larix gmelinii* (Miyamoto et al., 2020). Our findings indicate that pine species in Inner Mongolia may have a distinct EM fungal community. One explanation is climatic conditions; that is, the MAT and MAP in Inner Mongolia are lower compared with those in subtropical, tropical, and warm temperate areas; this may contribute to distinct EM fungal communities in Inner Mongolia, as MAT and MAP have been reported as strong drivers of EM fungal communities (Miyamoto et al., 2015). Soil development with different parent materials could give rise to the variations in soil physicochemical properties and thus may result in distinct microbial communities across different habitats (Alfaro et al., 2017; Sánchez-Marañón et al., 2017). Moreover, special species, such as *L. gmelinii* var. principis-rupprechtii, *Pinus sylvestris* var. mongolica, and *P. crassifolia* mainly distributed in Inner Mongolia, also could result in distinct EM fungal communities, which derived from host effect (Tedersoo et al., 2013).

The NMDS ordination combined with PerMANOVA suggested that host plants had a significant effect on the composition of the EM fungal community. Meanwhile, the environment fitting test and random forest analysis showed that host phylogeny, spatial distance, climatic variables, and some soil parameters significantly affected EM fungal community assembly, with host phylogeny being the strongest predictor. A similar finding has been reported in previous studies (Tedersoo et al., 2013; Wu et al., 2018; Wang Y.L. et al., 2019; Wang Z.H. et al., 2019), for example, studies involving Salicaceae, Betulaceae, and Fagaceae plant species all demonstrated that host plants were the most important determinant of EM fungal community assembly. In addition, 164 of 288 (56.94%) EM fungal OTUs existed in one plant species, Seventy-one OTUs (24.7%) appeared in two plant species, and only four OTUs were shared among the five pine species (Supplementary Figure 9). Meanwhile, the relative abundance of dominant lineages also showed a difference between host plants; for example, the relative abundance of *Tomentella–Thelephora* was significantly higher in *L. gmelinii* than in other hosts, and *Tricholoma* showed a significantly lower relative abundance in *P. crassifolia* than in other host plants (Supplementary Figures 10A,B). These results revealed that host plants had a significant impact on the EM fungal community. One explanation is the conservatism of the host phylogenetic niche (Losos, 2008); detailed, closely related plant species commonly showed more similarity than distantly related species in morphological and functional traits and appeared to share more similar fungal groups. A previous study indicated that host functional traits predicted 25 and 17% of the variation in root-associated pathogenetic and mycorrhizal fungal communities, whereas host effects were primarily due to host phylogeny (Wang Z.H. et al., 2019). Accordingly, our mantel tests showed that the dissimilarities of the EM fungal community increased significantly with the increase in plant phylogenetic distance (Mantel *r* = 0.335, *P* = 0.001), even after the control of variance in abiotic environmental factors and geographic distance (partial Mantel *r* = 0.228, *P* = 0.001), which further confirmed the important role of the host phylogenetic relationship in the community assembly of EM fungi. On the other hand, the preferences between plant and EM fungi generated over a long history of co-evolution could also contribute to the host effect on the fungal community, with all pine species and 54% of abundant EM fungal OTUs significantly preferring to certain partners, and 26% of plant–fungal pairs showing remarkably strong preferences in the SHWL dataset in the present study. Similar findings have been reported in previous studies involving EM fungi and Ericaceous fungi (Toju et al., 2016; Wu et al., 2018; Wang Y.L. et al., 2019).

Climate and soil parameters were found to affect EM fungal community in our study based on environmental fitting test and random forest analysis, and the effects of soil and climatic conditions on EM fungal communities have been intensively investigated in recent years (Miyamoto et al., 2015; Erlandson et al., 2016; Glassman et al., 2017). It has been well established that common fungal taxa grow well in proper habitats in soil nutrients, water content, and temperature (Cox et al., 2010; Miyamoto et al., 2018; Guo et al., 2020). For example, the frequency of some fungal taxa decreased with an increase in temperature, whereas some exhibited an opposite trend (Miyamoto et al., 2018; Koizumi and Nara, 2019); similarly, the population of some fungal taxa increased by additional nutrient inputs, whereas some were inhibited (Corrales et al., 2017). These findings suggested that different fungal taxa commonly occupied distinct niche positions. Overall, the effect of host plants, soil, and climatic variables on the EM fungal community assembly indicated a significant deterministic process in this study.

Apart from host plant, soil, and climatic variables, spatial distance also significantly predicted EM fungal community. The dissimilarity of the EM fungal community significantly increased by raising the geographic distance revealed by mantel and partial mantel tests, thus indicating that dispersal limitation is an essential determinant of the community assembly of EM fungi. Indeed, the low *m*-value (0.002) of the neutral model analysis also suggested that EM fungi experienced serious dispersal limitation and the EM fungal community assembly was well described by the stochastic process. In recent years, dispersal limitation has been widely accepted as an important driver of community assembly of soil- and root-associated fungi (Beck et al., 2015; Glassman et al., 2015; Wang Y.L. et al., 2019). One explanation is a spatial distance, which could impede the dispersal of fungal propagules (e.g., fungal individuals, spore, hypha) (Peay et al., 2010). In addition, the fungi varied in their dispersal abilities, and the order in the fungi that arrived at the habitat could give rise to variations in the fungal community (Peay et al., 2012). The significant effect of dispersal limitation on the EM fungal community suggested significant stochastic processes in shaping the community assembly of EM fungi, which was confirmed by the βNTI analysis that demonstrated the predominance of stochastic processes.

Overall, both deterministic and stochastic processes jointly controlled the community assembly of EM fungi associated with the five common pine species in Inner Mongolia, China, with the predominance of stochastic process based on the βNTI analysis. Among the variables investigated in the present study, host phylogeny was a better predictor than soil, climatic, and geographic factors in shaping the EM fungal community. It should be noted that these variables explained only a small proportion of the variation in the fungal community (20.15%, Figure 2B). This can be explained by the relatively few factors used in the analysis in our study; for example, soil pH, moisture, and other nutrient elements (potassium, organic matters, and available N) were absent here, whereas they have been proven to affect the EM fungal community in previous studies (Roy et al., 2013; Erlandson et al., 2016; Glassman et al., 2017). Moreover, the stochastic process derived from ecological drift and diversification were suspected to explain the community variation, as the βNTI analysis indicated the predominance of stochastic process in community assembly, but this needs to be investigated in our future study.

In summary, to our knowledge, this is the first time the EM fungal community associated with pine species in the semiarid and cold temperate forests in Inner Mongolia, China has been investigated. We found a high diversity of EM fungi in the five pine species; that is, 288 EM fungal OTUs that belonged to 31 lineages were reported in the present study. Host plant, soil, and climatic variables coupled with spatial distance jointly established the EM fungal community assembly, with host phylogeny being the best predictor. In addition, deterministic and stochastic processes concurrently shaped the community assembly of EM fungi, but stochastic processes prevailed. Our study shed light on the fungal diversity and community assembly processes of EM fungi associated with common pine species in northern China.

## Data Availability Statement

The original contributions presented in the study are publicly available. This data can be found here: The representative sequences of each EM fungal OTU were deposited in the European Nucleotide Archive (ENA) under study accession no. LR989273-LR989560. Information on EM fungi in the present study is shown in Supplementary Table 2.

## Author Contributions

Y-LW and Y-JF conceived and designed the study. XZ and YX performed the experiments. Y-LW analyzed the data and drafted the manuscript. XZ, S-MX, YX, and Y-JF performed the field sampling work. BB gave some suggestions for English improvement. Y-JF revised and approved the final manuscript. All authors contributed to the article and approved the submitted version.

## Conflict of Interest

The authors declare that the research was conducted in the absence of any commercial or financial relationships that could be construed as a potential conflict of interest.

## Footnotes

1 http://www.ehbio.com/test/venn/#/

## Supplementary Material

Supplementary Figure 1 Sampling sites selected in the Inner Mongolia, China. GH, Genhe; SHWL, Saihanwula; HGL, Huanggangliang; CF, Chifeng; HLH, Heilihe; WYD, Wangyedian; WLS, Wulashan; HLS, Helanshan.

Supplementary Figure 2 Maximum likelihood phylogram showing phylogenetic placement of pine species based on matK genes, using *Betula platyphylla* as an outgroup. Scale bar represents 5% sequence divergence.

Supplementary Figure 3 Ectomycorrhizal (EM) fungal operational taxonomic units (OTUs) ranked by relative abundance **(A)** and frequency **(B)**.

Supplementary Figure 4 Species accumulation curves of ectomycorrhizal (EM) fungal operational taxonomic units (OTUs) in five pine species. Lg, *Larix gmelinii*; Lgv, *Larix gmelinii* var. principis-rupprechtii; Pc, *Picea crassifolia*; Psv, *Pinus sylvestris* var. mongolica; Pt, *Pinus tabuliformis*.

Supplementary Figure 5 Krona chart of taxonomic affiliation of ectomycorrhizal (EM) fungi and their relative abundance. Inner circle represent higher taxonomic ranks and more detailed taxonomic ranks are presented in outer circles.

Supplementary Figure 6 Non-metric multidimensional scaling (NMDS) ordination of ectomycorrhizal fungal community composition based on Bray-Curtis distance of Saihanwula dataset (stress = 0.07). Ellipses delimit 95% confidence intervals around centroids for each pine species. Lg, *Larix gmelinii*; Lgv, *Larix gmelinii* var. principis-rupprechtii; Psv, *Pinus sylvestris* var. mongolica.

Supplementary Figure 7 Significant host, soil, climatic and spatial variables were fitted on the non-metric multidimensional scaling (NMDS) ordination of the EM fungal community composition.

Supplementary Figure 8 Mantel tests describing the correlations between ectomycorrhizal (EM) fungal community dissimilarity and plant phylogenetic distance **(A)** and geographic distance **(B)**.

Supplementary Figure 9 Venn diagram showing the shared and unique ectomycorrhizal fungal OTUs across the five pine species. Lg, *Larix gmelinii*; Lgv, *Larix gmelinii* var. principis-rupprechtii; Pc, *Picea crassifolia*; Psv, *Pinus sylvestris* var. mongolica; Pt, *Pinus tabuliformis*.

Supplementary Figure 10 Multiple comparison of the relative abundance of abundant ectomycorrhizal fungal lineage (>5% of total sequences). **(A)** tomentella-thelephora, **(B)** tricholoma, **(C)** sebacina, and **(D)** inocybe. Bars without shared letters indicate significant differences according to Dunn’s tests with Bonferroni adjustment at *P* < 0.05.

Supplementary Table 1 Sampling sites, plant species and environmental variables in present study.

Supplementary Table 2 Molecular identification of ectomycorrhizal fungi investigated in this study.

Supplementary Table 3 Accession number of matK gene of the pine species and outgroup species in present study.

Supplementary Table 4 Correlations of spatial, host, soil, and climatic variables with non-metric dimensional scale revealed by the environmental fitting test.
