# Supplementary material for: Fungal Diversity and Community Assembly of Ectomycorrhizal Fungi Associated With Five Pine Species in Inner Mongolia, China
Source: Front Microbiol. 2021 Mar 16;12:646821. doi: 10.3389/fmicb.2021.646821 (PMC8008119; doi:10.3389/fmicb.2021.646821)
Supplement: Supplementary Figure 1 — Sampling sites selected in the Inner Mongolia, China. GH, Genhe; SHWL, Saihanwula; HGL, Huanggangliang; CF, Chifeng; HLH, Heilihe; WYD, Wangyedian; WLS, Wulashan; HLS, Helanshan. [file Data_Sheet_1.docx]

Supplementary Materials for

**Fungal diversity and community assembly of ectomycorrhizal fungi associated with five pine species in the Inner Mongolia, China**

**Yong-Long Wang^1^, Xuan Zhang^1^, Ying Xu^1^, Busayo Joshua Babalola^2^, Si-Min Xiang^1^, Yan-Ling Zhao^1^ and Yong-Jun Fan^1*^**

^1^Faculty of Biological Science and technology, Baotou Teacher's College, Baotou, Inner Mongolia, China

^2^State Key Laboratory of Mycology, Institute of Microbiology, Chinese Academy of Sciences, Beijing 100101, China

*** Correspondence:** Yong-Jun Fan**:** [fanyj1975@163.com](mailto:fanyj1975@163.com)

# Supplementary Figures and Tables

## Supplementary Figures


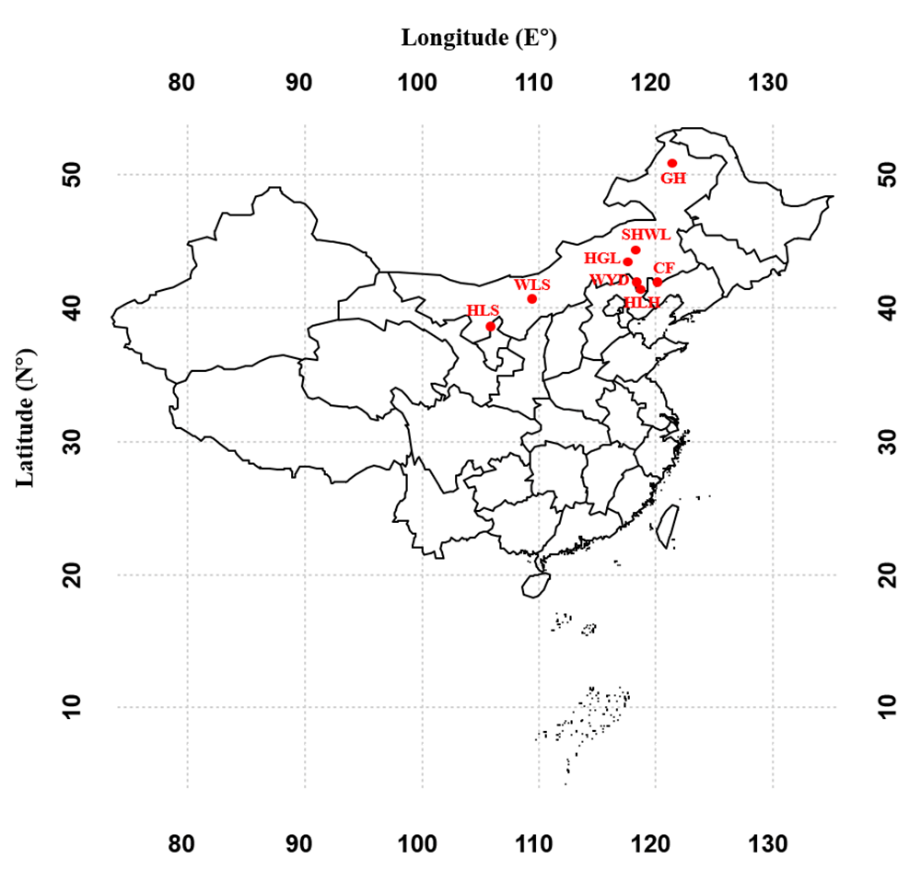


**Supplementary Figure 1.** Sampling sites selected in the Inner Mongolia, China. GH, Genhe; SHWL, Saihanwula; HGL, Huanggangliang; CF, Chifeng; HLH, Heilihe; WYD, Wangyedian; WLS, Wulashan; HLS, Helanshan.


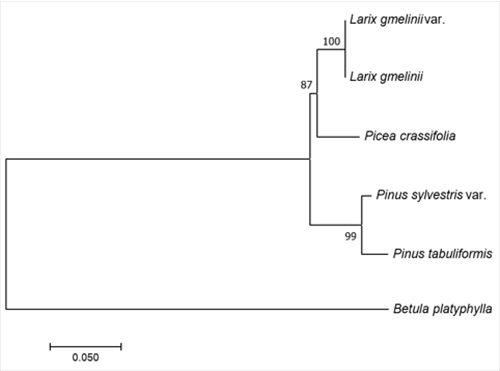


**Supplementary Figure 2.** Maximum likelihood phylogram showing phylogenetic placement of pine species based on matK genes, using *Betula platyphylla* as an outgroup. Scale bar represents 5% sequence divergence.


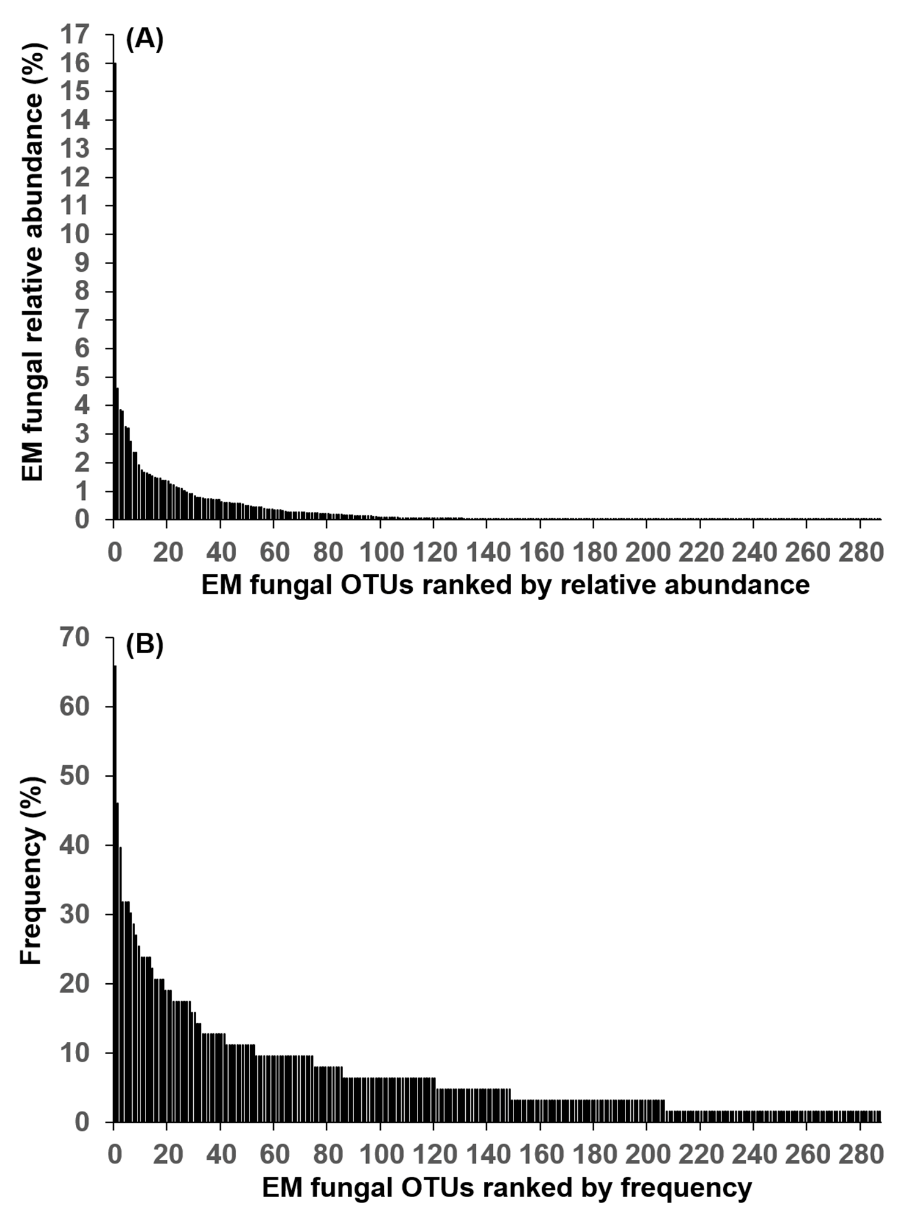


**Supplementary Figure 3.** Ectomycorrhizal (EM) fungal operational taxonomic units (OTUs) ranked by relative abundance **(A)** and frequency **(B)**.


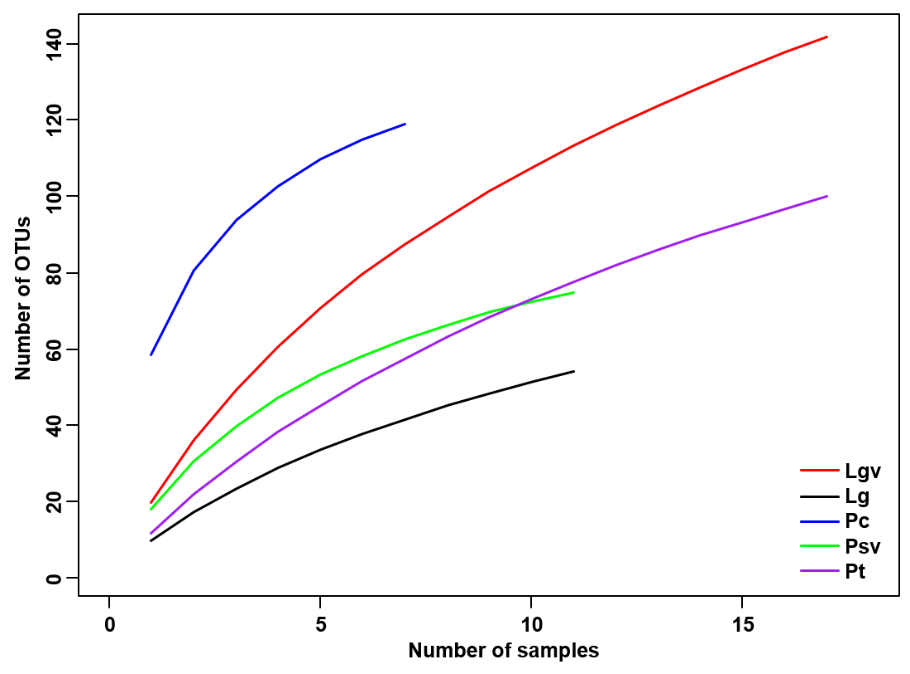


**Supplementary Figure 4.** Species accumulation curves of ectomycorrhizal (EM) fungal operational taxonomic units (OTUs) in five pine species. Lg, *Larix gmelinii*; Lgv, *Larix gmelinii* var. principis-rupprechtii; Pc, *Picea crassifolia*; Psv, *Pinus sylvestris* var. mongolica; Pt, *Pinus tabuliformis*.


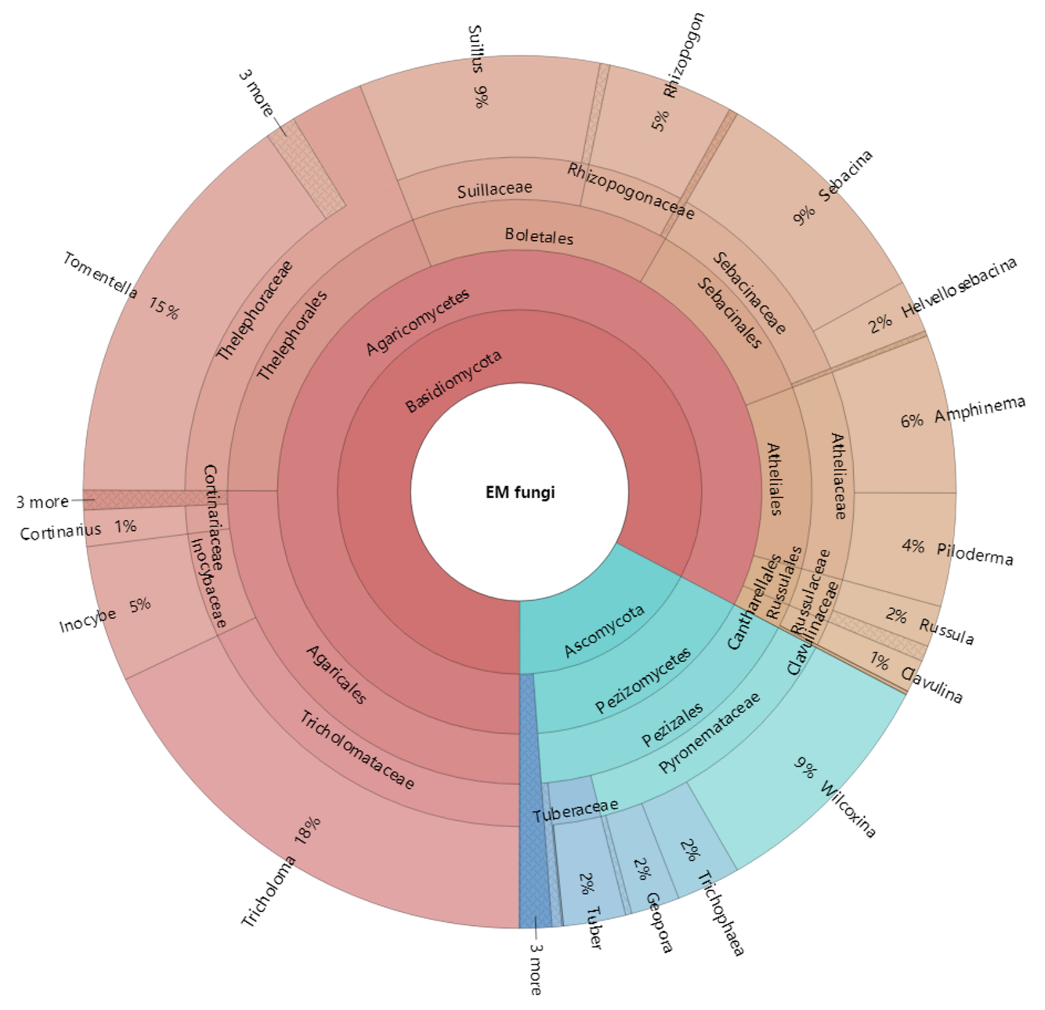


**Supplementary Figure 5.** Krona chart of taxonomic affiliation of ectomycorrhizal (EM) fungi and their relative abundance. Inner circle represent higher taxonomic ranks and more detailed taxonomic ranks are presented in outer circles.


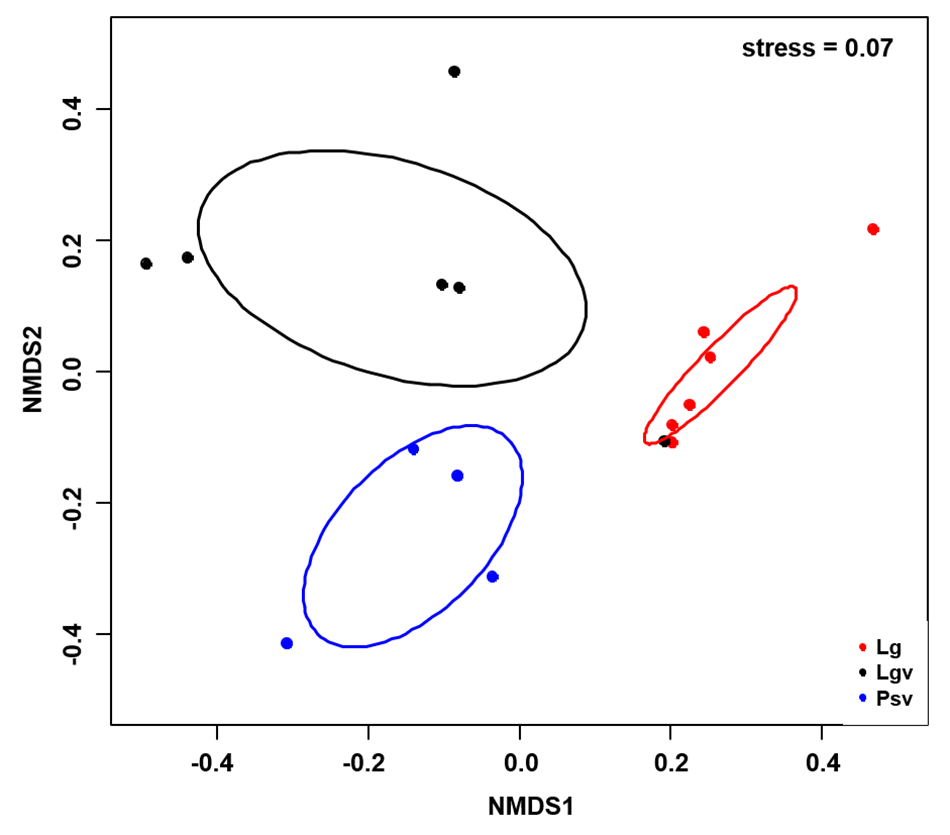


**Supplementary Figure 6.** Nonmetric multidimensional scaling (NMDS) ordination of ectomycorrhizal fungal community composition based on Bray-Curtis distance of Saihanwula dataset (stress = 0.07). Ellipses delimit 95% confidence intervals around centroids for each pine species. Lg, *Larix gmelinii*; Lgv, *Larix gmelinii* var. principis-rupprechtii; Psv, *Pinus sylvestris* var. mongolica.


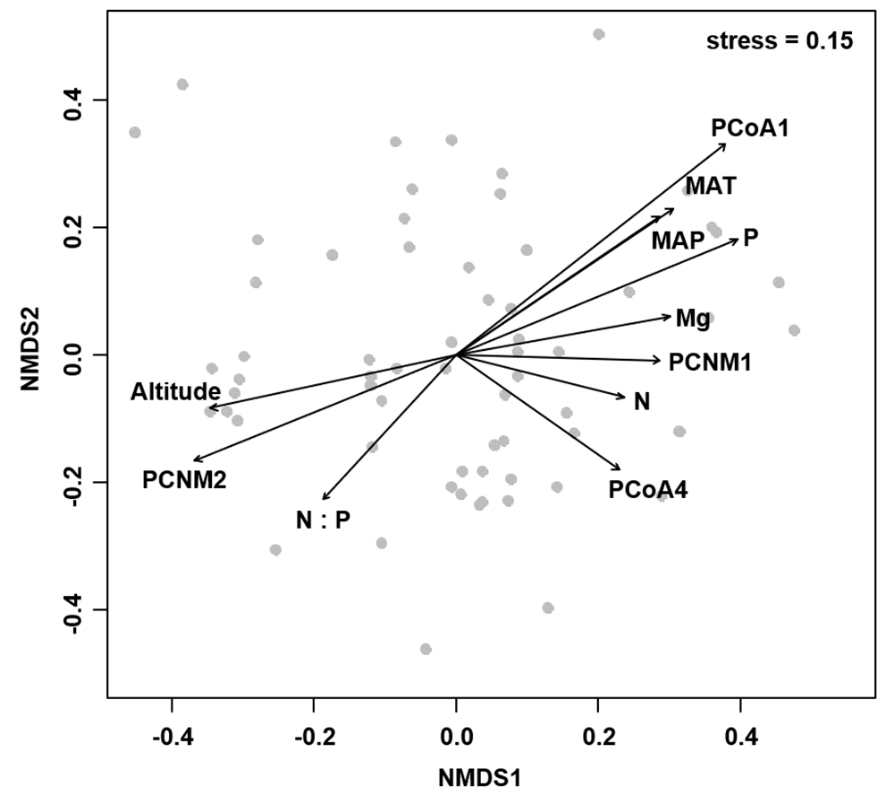


**Supplementary Figure 7.** Significant host, soil, climatic and spatial variables were fitted on the non-metric multidimensional scaling (NMDS) ordination of the EM fungal community composition.


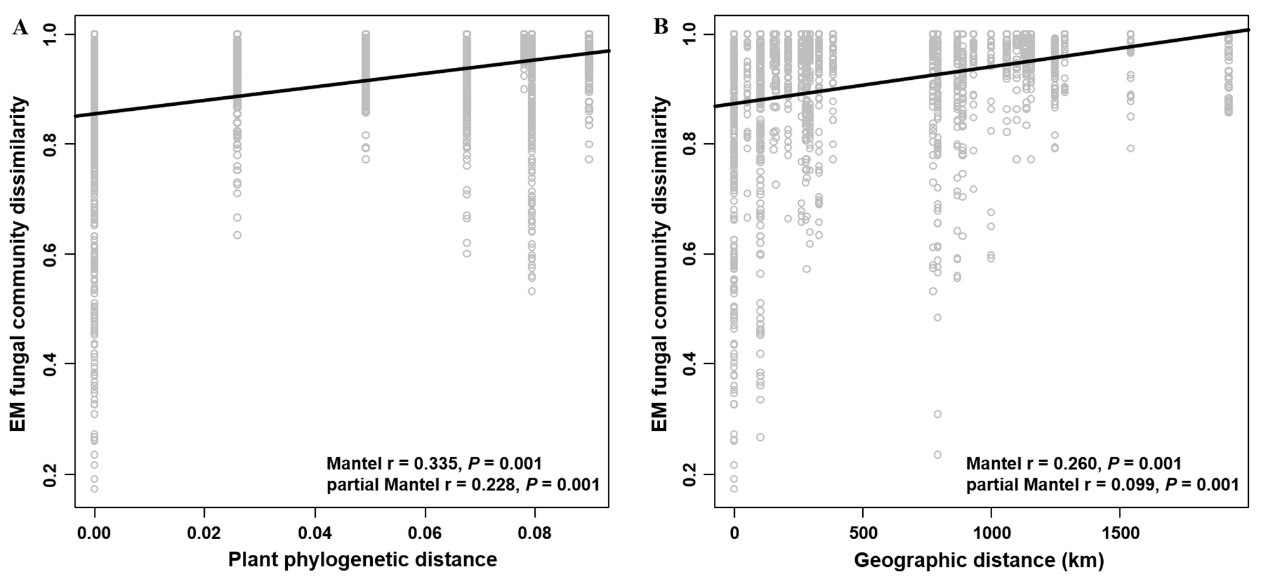


**Supplementary Figure 8.** Mantel tests describing the correlations between ectomycorrhizal (EM) fungal community dissimilarity and plant phylogenetic distance (A) and geographic distance (B).

**
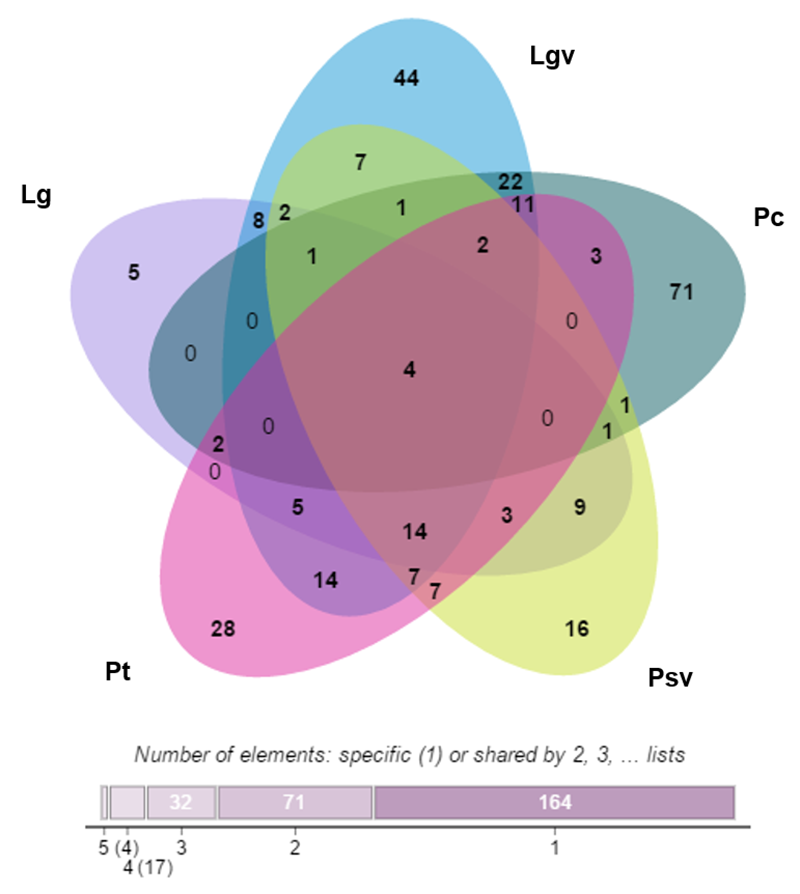
**

**Supplementary Figure 9.** Venn diagram showing the shared and unique ectomycorrhizal fungal OTUs across the five pine species. Lg, *Larix gmelinii*; Lgv, *Larix gmelinii* var. principis-rupprechtii; Pc, *Picea crassifolia*; Psv, *Pinus sylvestris* var. mongolica; Pt, *Pinus tabuliformis*.

**
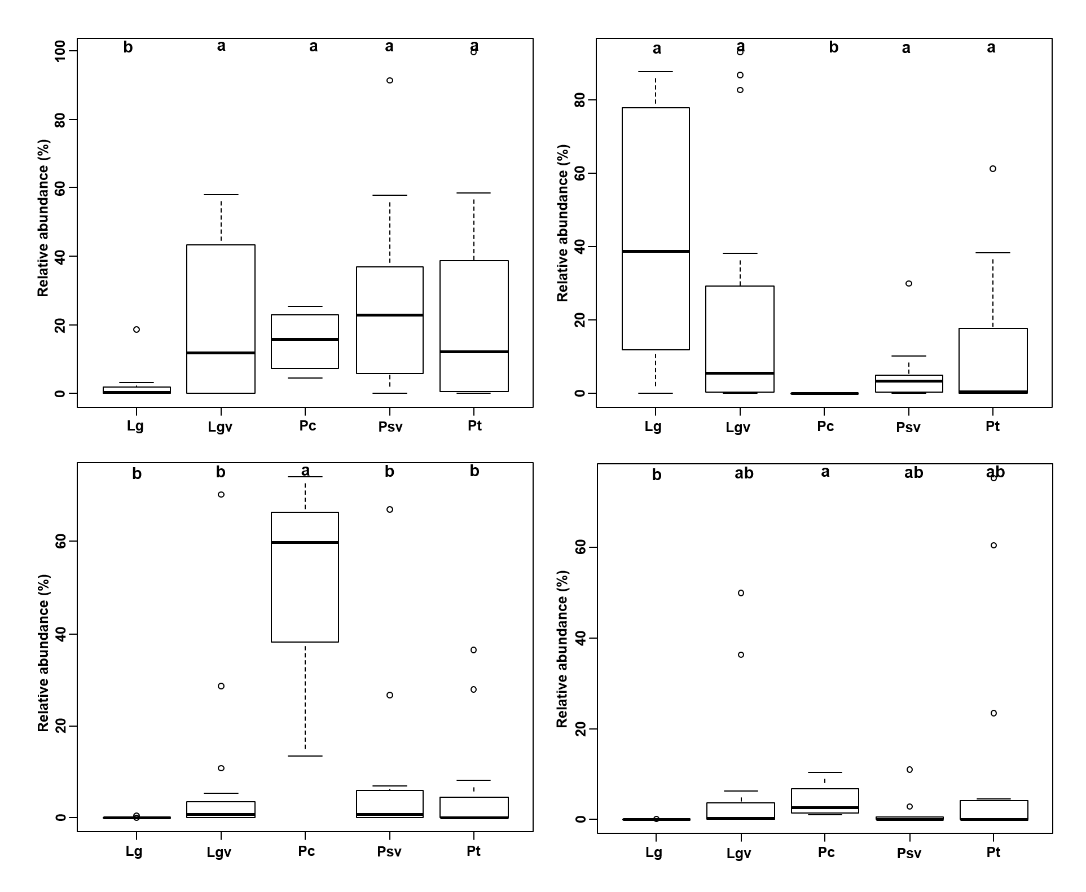
**

**Supplementary Figure 10.** Multiple comparison of the relative abundance of abundant ectomycorrhizal fungal lineage (> 5% of total sequences). **(A)** /tomentella-thelephora, **(B)** /tricholoma, **(C)** /sebacina and **(D)** /inocybe. Bars without shared letters indicate significant differences according to Dunn’s tests with Bonferroni adjustment at *P* < 0.05.

## Supplementary Figures

**Supplementary Table 1.** Sampling sites, plant species and environmental variables in present study.

**Supplementary Table 2.** Molecular identification of ectomycorrhizal fungi investigated in this study.

| **Supplementary Table 3.** The accession numbers of pine species and outgroup species used in present study. | |
| --- | --- |
| Plant species | Accession number |
| *Pinus tabuliformis* | JF955534 |
| *Pinus sylvestris* var. mongolica | JF955489 |
| *Larix gmelinii* var. principis-rupprechtii | AY391403 |
| *Larix gmelinii* | MH660045 |
| *Picea crassifolia* | JF955434 |
| *Betula platyphylla* | AY372023 |

| **Supplementary Table 4.** Correlations of spatial, host, soil and climatic variables with non-metric dimensional scale revealed by environmental fitting test. | | | | | |
| --- | --- | --- | --- | --- | --- |
| Variable | NMDS1 | NMDS2 | *R*^2^ | Pr(>r) | significance |
| PCNM1 | 0.99944 | -0.0334 | 0.1513 | 0.009 | ** |
| PCNM2 | -0.9095 | -0.4158 | 0.3016 | 0.001 | *** |
| PCNM3 | 0.99829 | 0.05843 | 0.0198 | 0.564 |  |
| PCoA1 | 0.75373 | 0.65719 | 0.4684 | 0.001 | *** |
| PCoA2 | -0.7378 | 0.67499 | 0.078 | 0.092 |  |
| PCoA3 | -0.6238 | -0.7816 | 0.0168 | 0.594 |  |
| PCoA4 | 0.78388 | -0.6209 | 0.1583 | 0.004 | ** |
| Altitude | -0.971 | -0.2393 | 0.2345 | 0.001 | *** |
| MAT | 0.80046 | 0.59939 | 0.2688 | 0.001 | *** |
| MAP | 0.79651 | 0.60462 | 0.2384 | 0.001 | *** |
| N | 0.96132 | -0.2754 | 0.1114 | 0.023 | * |
| P | 0.91059 | 0.41331 | 0.3499 | 0.001 | *** |
| N : P | -0.6368 | -0.771 | 0.1612 | 0.009 | ** |
| Ca | 0.23364 | -0.9723 | 0.0138 | 0.648 |  |
| Mg | 0.98128 | 0.1926 | 0.174 | 0.007 | ** |
| PCNM, principal coordinates of neighbor matrices; PCoA, principal coordinates of analysis; MAT, mean annual temperature; MAP, mean annual precipitation; N, soil total nitrogen; P, soil total phosphorus; Ca, soil total calcium; Mg, soil total magnesium. | | | | | |
